# The Microbe of Cancer

**Published:** 1887-08

**Authors:** 


### THE MICROBE OF CANCER.

Dr. Domingo Freire, who claims to have discovered the preventive inoculation of cholera, has recently attempted to demonstrate the microbic nature of cancer.

Freire examined the blood of a cancerous woman and discovered in it zooglea capable of cultivation in gelatin kept at a temperature of from 37° to 40° C., and developing into bacilli 0.011 m. in length by 0.002 in breadth, enlarged at one extremity and resembling the bacilli of typhoid fever. In the cultivation he observed also spores, zooglea and collections of small bacilli which appeared to be the various stages in the development of the bacilli. Without determining whether he had cultivated one or many varieties of bacteria, Freire concluded that he had found the specific microbe of cancer, and that this microbe passes through two stages in its evolution. The first is represented by the micrococci in zooglea and the second, more advanced, by the bacilli which are capable of living only outside of the blood, but are found in the cancerous juice which covers the ulcer. The writer does not mention the cultivation of these latter bacilli, and has apparently not determined whether or not they will produce bacilli like the former. To explain the cancerous cachexia he examined the urine of cancerous patients and discovered a ptomaine, which is very poisonous to birds, causing death with convulsions. But the recent experiments of Gautier and Bouchard show that even normal urine contains poisonous alkaloids, and the progressive development of the tumor in the organs essential to life would explain the cachexia. Freire then inoculated guinea pigs with the cultivation. He produced a slight wound and injected into it a solution containing the germs. At the end of a month one animal died and the autopsy revealed the presence of a tumor about the size of a hen's egg, located in the left iliac fossa below the fold of the peritoneum. The tumor was soft, and on pressure exuded a viscid substance of about the consistency of brain. This is the most characteristic of all the experiments reported by the author. Microscopic examination of this tumor, as well as of the others obtained, demonstrated that they were encephaloid carcinomata. There were agglomerations of giant cells, and there was no doubt in Freire's mind that the tissue was really cancerous and produced by the inoculation. The experimenter also succeeded in attenuating the cancerous virus by passing it through a number of birds, and the animals that had been inoculated in this manner acquired immunity from the stronger preparations. The investigation demonstrated to a certain extent the nature of cancer, and indicates a method of treatment for this terrible affection, hitherto looked upon as incurable.

It will be interesting to refer here to the experiments of Galippe and Landouzy, who communicated their results to the *Sociedad de Biologia*. These writers announce that they have discovered micro-organisms in fibroid tumors of the uterus and in ovarian cysts. The parasites are of three varieties, 1, sphero-bacteri in diplococci and in chains; 2, other micrococci, less common, smaller and forming cancer; 3, bacilli, isolated or united, forming threads. They succeeded in cultivating these microbes in warm gelatin, but did not make inoculation, and in consequence no conclusion as to their pathogenetic properties have been reached. These authors compare these tumors with those found at the roots of the teeth, studied by Malassey, and express the opinion that they are both due to the penetration of microbes from the neighboring cavities, the buccal and vaginal. Myomata of the uterus are more frequent than myomata of all other organs combined, and ovarian and radiculo-dental cysts are those most frequently developed. Here, then, is a new field opened up to bacteriologists. Galippe and Landouzy believe that many animal tumors are absolutely identical in their origin with many vegetable tumors known to be of parasitic origin. This is a legitimate deduction in general pathology, which will sooner or later be demonstrated. The idea of a *neoplastic diathesis*, advanced by Verneuil, is thus reduced to a question of soil and auto-inoculation.—*Revista Med., Quirurg. de Buenos Aires.*

